# Ensuring the Quality and Appropriate Use of Hand Sanitizers During the COVID-19 Pandemic: Suggestions and Recommendations With the Role of the Pharmacist

**DOI:** 10.1017/dmp.2021.55

**Published:** 2021-02-16

**Authors:** Muhammad Hammad Butt, Abrar Ahmad, Shahzadi Misbah, Tauqeer Hussain Mallhi, Yusra Habib Khan, Khayal Muhammad, Zafar Iqbal

**Affiliations:** 1 Faculty of Pharmacy, University of Central Punjab, Lahore, Pakistan; 2 Department of Clinical Pharmacy, College of Pharmacy, Jouf University, Sakaka, Al-Jouf Province, Kingdom of Saudi Arabia; 3 Department of Clinical Pharmacy, Faculty of Pharmacy, Near East University, Nicosia, North Cyprus, Turkey; 4 Department of Pharmaceutical Services, Armed Forces Hospital, King Abdulaziz Air Base, Dhahran, Kingdom of Saudi Arabia

**Keywords:** hand Sanitizers, COVID-19, SARS-CoV-2, quality, recommendations

Currently, there is no specific treatment for coronavirus disease (COVID-19), and the availability of the newly developed vaccine is limited. Precautionary measures, such as wearing masks, social distancing, handwashing, surface disinfecting, and using hand sanitizers, have remained the only effective measures during the ongoing pandemic.^1,2^ Of these, quality, availability, and appropriate use of hand sanitizers hold paramount importance and consideration. The World Health Organization (WHO) recommends 2 alcohol-based formulations to lessen the spread and infectivity of the novelty virus. Kratzel et al. investigated the efficacy of both the WHO recommended formulations and found them equivalent in terms of efficacy for COVID-19.^3^

Unfortunately, to date, 7593 cases of hand sanitizer exposure in children under the age of 12 years have been reported.^4^ According to the CNN news reported in New Mexico, out of 7 people who ingested methanol-based hand sanitizers, 3 died, 3 were in critical condition, and 1 experienced permanent blindness.^5^ The Food and Drug Administration (FDA) has issued a warning for the general public to avoid sanitizers from unauthentic companies (namely, Mexican company) due to the risk of toxic effects among users.^6^

Following the WHO recommendations on the use of hand sanitizers as a preventive measure for COVID-19, the demand for these products surged around the globe, resulting in their shortage and unavailability. Pakistan has reported an increased demand of hand sanitizers up to 3000 tons from its regular demand of 1200 tons.^7^ A similar surge was observed in other developing and developed countries. It is pertinent to mention that the announcement by the WHO on the effectiveness of sanitizers caused stock shortages and price hikes. To overcome the shortage and to meet the increasing demand, many pharmaceuticals and chemical, perfume, and beverage industries have started the production of hand sanitizers.

The increased demand for hand sanitizers resulted in a mass production, which subsequently led to the availability of substandard products with varying concentrations of alcohol in the consumer market. It must be noted that the use of substandard hand sanitizers can be linked to numerous adverse events involving permanent blindness, gastrointestinal irritations, portal vein embolism, mild mucosal irritation, eczema, skin irritation, respiratory problem, resistance to antibiotics, hormonal problems, depression, hypothermia, hypertension, ketoacidosis, alcohol poisoning, and in severe cases – death.^4^ We believe that health authorities should run parallel maneuvers to ensure the quality and standards of hand sanitizers as a component of a vigorous COVID-19 containment campaign.

## Suggestion and Recommendation With the Role of the Pharmacist

To overcome or fulfill the demand for hand sanitizers, controlling the price hike, and lessening the hazards of these products, we are inclined to share a few suggestions to curb the situation of COVID-19. The industrial pharmacist can play the role in providing standard production of hand sanitizers by maximizing the production, quality control, assurance, and compliance with official guidelines as provided by the WHO, FDA, and pharmacopeias. Drug regulatory and health authorities should ensure the standard production and the availability of hand sanitizers in hospitals, pharmacies, health care centers, and high-risk vicinities at affordable prices. Moreover, implementation monitoring strategies and ensuring the regulatory practices are of utmost importance during the current circumstances. In cases of shortage, priority should be given to the high-risk areas, and supply should be maintained. In addition, community pharmacists can ensure the safe and effective use of these products and can optimize the preparation of hand sanitizers within the pharmacy premises by taking advantage of small-scale manufacturing. Moreover, they are much aware on the importance of complying with the recommended specifications and guidelines. These practices would not only be translated into a drastic reduction of the cost to the public, but also will aid in the provision of standard and quality products to the consumers. Furthermore, a community pharmacist is an accessible professional to the community and can educate the public on the safe use of hand sanitizers, hereby ensuring the safety on the community level. Community pharmacists counsel people to avoid touching the facial area after using hand sanitizers, as exposure can cause skin irritation. They can also counsel the public regarding the storage of hand sanitizer at room temperature, preferably at cool places, and keeping the hand sanitizer out of the reach of children. The pharmacist can also play the role in providing post-ingestion management of hand sanitizers. Pharmacists can assist in the case management of adverse events following ingestion of hand sanitizers either directly or indirectly by providing the swift information on treatment protocols. Pharmacists as health care professionals can play a major role in current crises by providing awareness, education, and guidance related to appropriate selection, buying and use of hand sanitizer, monitoring any substandard hand sanitizer brands, which can provide a threat to public health instead of helping curb the COVID-19. Taken together, collaborative efforts of pharmacists with health authorities will mitigate the harms and burden of sanitization associated adverse events, for which none of the countries is readily prepared amid an overwhelming health care system.
